# Resilience in cancer patients and how it correlates with demographics, psychological factors, and lifestyle

**DOI:** 10.1007/s00432-022-04480-6

**Published:** 2022-11-18

**Authors:** Lara Festerling, J. Buentzel, L. Fischer von Weikersthal, C. Junghans, B. Zomorodbakhsch, C. Stoll, F.-J. Prott, S. Fuxius, O. Micke, A. Richter, D. Sallmann, J. Huebner, Catalina Hoppe

**Affiliations:** 1 Klinik für Innere Medizin II, Hämatologie und Internistische Onkologie, Universitätsklinikum Jena, Friedrich-Schiller Universität Jena, Am Klinikum 1, 07747 Jena, Germany; 2 Klinik für HNO-Erkrankungen, Kopf-Hals-Chirurgie, Interdisziplinäre, Palliativstation, Südharz Klinikum Nordhausen, Dr.-Robert-Koch-Straße 39, 99734 Nordhausen, Germany; 3 Gesundheitszentrum St. Marien GmbH, Praxis Für Hämatologie und Internistische Onkologie, Mariahilfbergweg 7, 92224 Amberg, Germany; 4 Paracelsus Klinik am Schillergarten Bad Elster, Martin-Andersen-Nexö-Str. 10, 08645 Bad Elster, Germany; 5 üBAG/MVZ Onkologische Kooperation Harz GbR, Kösliner Straße 14, 38642 Goslar, Germany; 6 Klinik Herzoghöhe Bayreuth, Kulmbacher Straße 103, 95445 Bayreuth, Germany; 7 Strahlentherapie am St. Josef Krankenhaus, Beethovenstraße 20, 65189 Wiesbaden, Germany; 8 Onkologische Schwerpunktpraxis Heidelberg, Kurfürsten-Anlage 34, 69115 Heidelberg, Germany; 9 Franziskus Hospital, Kiskerstraße 26, 33615 Bielefeld, Germany; 10 Inselsberg Klinik Wicker GmbH & Co. OHG, Fischbacher Str. 36, 99891 Bad Tabarz, Germany; 11 REGIOMED REHA-Klinik Masserberg GmbH, Hauptstraße 18, 98666 Masserberg, Germany

**Keywords:** Resilience, Cancer, Multicentric cross-sectional study, Self-efficacy, General life satisfaction, Sense of coherence

## Abstract

**Background:**

Being diagnosed with cancer is challenging. Many patients wish to be actively involved in treatment and contribute to therapy, but the patients’ coping abilities and desire for involvement differ. The individual level of resilience seems to play a major role. Our study aims to learn more about the associations of resilience and factors as demographics and psychological factors.

**Methods:**

This multicentric cross-sectional study was conducted in ten oncological centers in Germany in summer 2021. The questionnaire collected information on demographics, resilience, self-efficacy, general satisfaction with life, and sense of coherence. Considered lifestyle-aspects were diet and physical activity. 416 patients were included in the analyses.

**Results:**

A moderate mean resilience score was achieved (*M* = 69). Significant correlations in demographics were found for resilience and education (*r* = 0.146, *p* = 0.003), income (*r* = 0.205, *p* = 0.001), and time since receiving diagnosis (*r* = − 0.115, *p* = 0.021). Resilience and self-efficacy correlated on a high level (*r* = 0.595, *p* < 0.001), resilience and sense of coherence, and resilience and general satisfaction with life in a moderate way (*r* = 0.339, *p* < 0.001; *r* = 0.461, *p* = 0.001).

**Conclusions:**

Resilience portrays an important aspect in cancer treatment. Detecting patients at risk, stabilizing, or improving resilience are important to focus on and strengthen them accordingly. Possible negatively influencing factors (e.g., low self-efficacy) need to be considered. Factors affecting resilience but difficult to influence, as educational background, should be screened for. Also, the combination of low resilience and low income seems to describe a vulnerable patient group.

## Introduction

Receiving a diagnosis for cancer is a difficult situation to deal with for most people and affects many aspects of their and their next one’s life. Although it is undoubted that this diagnosis is challenging, some patients cope better than others. One factor that plays a crucial role in this coping ability is the patient’s level of resilience. Resilience is defined as an individual’s ability to cope with distress and adapt to challenging events, such as the diagnosis of a life-threatening disease (Davydov et al. 2010). It describes the ability to restore a stable mental and physiological status in or after burdensome events, such as the death of a close relative, loss of workplace and others. Resilient people seem to be able to reflect on their positive and negative emotions better than less resilient patients, which helps to restore resources and react more flexible (Tugade et al. 2004).

According to different authors, the extent of an individual’s resilience is to a certain degree, trainable and learnable, as well as to some degree determined by factors such as genetics (Davydov et al. 2010; Connor and Zhang xxxx). Literature states that resilience can be described as a dynamic process (VanMeter and Cicchetti 2020; APA 2020) and is not a trait the individual is born with, but it develops throughout life based on experiences and learning (BZgA 2021).

If one looks at previous studies, one finds that resilience is often associated with mental well-being (Färber and Rosendahl 2018). Resilience is brought into close relation with the individual being more optimistic, see more things as an opportunity to benefit from and also a greater emotional consciousness (Babic et al. 2020). Seiler and Jenewein (2019) showed that resilience performs as a protective factor against psychological distress and is closely related to a patient’s optimism. Since self-efficacy plays an important role in the concept of resilience (Wu et al. 2021), both these terms are closely linked to each other (Schumacher et al. 2014). As self-efficacy is considered to be a mediating factor for resilience and its effect on how people cope with illness (Karademas et al. 2022). Also, satisfaction with life, spirituality and as shown by Seiler and Jenewein (2019), sense of coherence can be closely connected to resilience and are therefore interesting factors for examination. Cancer affects all aspects of life and not only the patients’ health status, and hence, we considered factors such as daily activity, diet, and life satisfaction to be as important to integrate and gain information about. Especially because not much data exists on physical activity and resilience. A positive correlation is reported by a study by Schumacher et al. (2014), another by Eicher et al. (2015). They show that a higher resilience is connected to better physical functioning. Actively including patients with cancer in the treatment is not only recommendable, but also often wished-for. Most of these patients are highly interested in diets and some in physical activity (Braun et al. 2019).

Due to the significance of resilience, identifying patients at risk of a lower level and strengthening resilience should certainly be a priority. The intention is to find out what certain characteristics or criteria may be a target for cancer patients to improve or preserve their resilience in respect of coping with their disease and supporting patients in actively getting involved in treatment. According to Ludolph et al. (2019), interventions based on therapies for positive psychology and supportive groups or behavioral therapies show the most beneficial results in respect of promoting resilience in cancer patients.

Collecting information about less resilient cancer patients will give us the tools for identifying these people ahead of time. Above identifying, making tools available for patients to stabilizing, ideally strengthen and preventing attenuation of their resilience is a major goal. Based on this, it would again be possible to create a guide or handout recommendations for doctors and other involved care attendants, have this evaluated at different oncological centers among Germany and expand its use accordingly.

To pursue this, it is necessary to learn more about the associations of resilience. This study focuses on how resilience is connected to different demographics, other psychological factors, and diverse aspects of lifestyle.

## Patients and methods

### Study design

This prospective multicentric cross-sectional study was conducted in ten oncological centers in Germany. Data acquisition was carried out from March 2021 until July 2021.

### Study participants

The questionnaire was distributed in oncological centers (six oncological departments of hospitals, two rehabilitation clinics, and two oncological offices) in Germany to outpatient cancer patients. Inclusion criteria were a diagnosis of and active cancer treatment and a sufficient knowledge of the German language to answer the questions independently. Exclusion criteria were age below 18 years. As this study focuses on resilience, an additional criterium was that the patient needed to have a valid resilience score. The participants were asked to answer the questionnaire anonymously in a print version by pen.

### Questionnaire

The questionnaire consisted of two parts. The first part contained questions regarding the demographics, such as gender, age, type of cancer, and time since first diagnosis.

The second part consisted of different, established, and validated questionnaires.

1. We used the RS-13 short scale to file the patients’ resilience. The RS-13 questionnaire by Leppert et al. (2008) to file the participants’ resilience consists of 13 items with two subcategories acceptance and competence. This short version is based on the RS-25 by Schumacher et al. (2005) and has a sufficient re-test reliability of 0.62. The RS-13 contains a 7-point Likert scale (1 = “I don’t agree at all”; 7 = “I fully agree”) and exhibits an excellent internal reliability (Cronbach’s *a* = 0.9). Values from 13 to 66 are considered to be “low”, 67–72 points are a “moderate” level and 73–91 stand for a “high” level of resilience. For patients who skipped one item, we substituted the missing value by the stated values’ mean. All other questionnaires that missed more than one item were considered to be invalid and not included in further evaluation as no valid score could be calculated.
2. The general life satisfaction short scale L-1 is a 10-point Likert-Scale (Beierlein et al. 2013). This short scale shows a re-test-reliability of 0.67. In comparison with the multi-item short scale (Diener et al. 1985), it also shows a high positive correlation (*r* = 0.74) (Beierlein et al. 2021).
3. To gather information about the patients’ perceived self-efficacy, we used the “Allgemeine Selbstwirksamkeit-Kurzskala/general self-efficacy short scale” (ASKU), a short scale with a sufficient internal consistency between 0.81 and 0.86. Other studies showed a high positive correlation with the participants’ self-worth, their general life satisfaction, and internal control conviction (Beierlein et al. 2013). All items are collected with a 5-point Likert scale, resulting in values between 1 and 5.
4. The Sense of Coherence SOC-L9 questionnaire files the patients’ sense of coherence with the three components of meaningfulness, manageability, and comprehensibility (Schumacher et al. 2000). The employed short scale shows an excellent correlation with longer versions such as the SOC-29 (*r* = 0.94), as well as an adequate internal consistency (Cronbach’s *a* = 0.87). In case of a missing value, it was substituted by the individual mean value the patient stated on this questionnaire.
5. Daily activity was enquired by a Likert scale with three options, 0 standing for less than 10 min, 1 for a time between 11 and 30 min of activity, 2 for an activity level between 31 and 60 min a day and 3 signifying an activity level of more than 60 min. The patients were asked to give information about their activity level before and after the diagnosis.
6. For elaboration on the patients’ dietary habits and active investment, the Adolescent Food Habits Checklist (AFHC) we used in this survey was shortened (12 items instead of 23 items) and translated. It collects information on the active investment in their diet and general dietary habits of the patients. Due to the original questionnaire containing doublings, we shortened it to 12 items to keep the questionnaire clear.

### Statistical analysis

Statistical analyses were conducted using Statistical Package for Social Science (IBM SPSS) version 27. For pairwise correlation analyses values for both, resilience and the specific variable, had to exist.

Adapted to the scale of measurement in question, Pearson’s correlation coefficient or Spearman’s rank correlation coefficient was used to assess relationships between demographic factors and resilience. For assessment of group differences in resilience analysis of variance *t* tests have been carried out. For variables with more than two groups, ANOVA with Bonferroni was performed, for samples that consisted of two groups only, independent sample t tests were used. Group differences for age were assessed by the Mann–Whitney *U* test. *p* < 0.05 was considered to be significant.

## Results

### Demographics

A total of 451 patients out of 10 oncological centers and offices in Germany took part in this study. The RS-13 questionnaire was filled by 416 participants (92.2%) who were included in further evaluation.

The participants mean age was 62.3, reaching from 33 to 85 years; 268 (66.0%) were female and 138 (34.0%) were male.

Further detailed information on demographical data can be found in Table 1.

**Table 1:** Demographic data (*N* = 416)

Data	*N*	(%)
Marital status		
Married	282	67.8
Divorced	44	10.6
Widowed	35	8.4
Relationship	29	7.0
Single	26	6.3
Financial coping without income		
1–6 months	141	36.0
6–12 months	94	24.0
More than 12 months	157	40.1
Level of education		
No degree	9	2.2
8/10th grade	260	63.6
Abitur	31	7.6
University/College	109	26.7
Religion		
Christian	233	57.0
None	172	42.1
Muslim	3	0.7
Other	1	0.2
Type of cancer		
Breast	146	37.1
Colorectal	49	12.4
Head–neck	45	11.4
Gastrointestinal	31	7.9
Leukemia, lymphoma	29	7.4
Prostate	21	5.3
Lung	21	5.3
Gynecological	21	5.3
Other urogenital	6	1.5
Others	25	6.3
Time since diagnosis		
Less than 1 year	208	51.5
1–3 years	113	28.0
3–6 years	41	10.1
More than 6 years	42	10.4

### Resilience

In the population, a mean resilience score of 69 was achieved, which lies in the moderate range of the instrument. One hundred and ninety-five patients (46.9%) scored a high resilience value, and 16.3% (*N* = 68) had a moderate one, while 36.8% (*N* = 153) scored a low value. Results of the correlation analyses are shown in Table 2 and are explained below.

**Table 2:** Correlations of resilience and reported variables

Variable	*N*	Correlation coefficient	*p*
Age	412	r = 0.036	> 0.05
Gender	406	η = 0.023	> 0.05
Marital status	416	η = 0.088	> 0.05
Financial coping without income	392	r = 0.205	0.001
Level of education	409	r = 0.146	0.003
Religion	409	η = 0.059	> 0.05
Type of cancer	394	η = 0.126	> 0.05
Time since diagnosis	404	r = − 0.115	0.021
General life satisfaction	381	r = 0.461	0.001
Self-efficacy	405	r = 0.595	0.001
Sense of coherence	416	r = 0.339	0.001
Dietary habits	404	r = 0.117	0.018
Daily activity	382	r = 0.142	0.005

(Differences in *N* are due to missing information given by the patients)

#### Resilience and demographic data

There were no correlations between resilience and age, gender, marital status, and none between resilience and religious affiliation. Also, no significant difference could be observed for the type of cancer (*p* > 0.05).

Resilience was weakly correlated to a higher level of education (*r* = 0.146, *p* = 0.003) and a better income (*r* = 0.205, *p* = 0.001). In contrast, a long-standing diagnosis is correlated with lower resilience (*r* = − 0.115, *p* = 0.021).

#### Resilience and lifestyle

Lifestyle in this context summarizes the factors dietary habits according to the AFHC and daily activity.

The Adolescents Food Habits Checklist Score showed a mean score of 7.8 out of 12 possible points. The food habit checklist and resilience were positively and significantly correlated (*r* = 0.117, *p* = 0.018).

Results from the activity-questionnaire showed that the majority (61.8%) achieved an average current daily activity of at least 31–60 min. The patient’s current daily activity was positively correlated to their resilience score (*r* = 0.142, p = 0.005). When comparing the mean value for daily level of activity now (*M* = 2.02), to what the patients stated to have had before their diagnosis (*M* = 2.63), a slight decrease of the mean value could be observed (*t*(363) = 11.98, *p* < 0.001).

#### Resilience and psychological factors

As psychological factors we summarized general life satisfaction, self-efficacy, and sense of coherence. A moderately high correlation for resilience and general life satisfaction was found (*r* = 0.461, *p* = 0.001) with a mean value of 6.38.

The mean value of self-efficacy was 3.92. The correlation between resilience and self-efficacy was significant on a high level (*r* = 0.595, *p* < 0.001).

Concerning sense of coherence, a mean score of 32.17 was achieved. This score lies in the middle range of the scale. The perceived resilience and the patients’ sense of coherence were found to be correlated on a significant moderate level, showing that a higher sense of coherence goes along with a higher resilience (*r* = 0.339, *p* < 0.001).

## Discussion

In our cross-sectional study, we have shown a high correlation between resilience and self-efficacy, while between resilience and general life satisfaction, there was a moderate correlation as well as between resilience and sense of coherence. In contrast, the financial status, obtained level of education, time since diagnosis, dietary habits, and level of daily activity only show less strong correlations with resilience.

### Resilience and demographic data

Resilience and age do not show correlation in our study population, opposed to literature which reports it to be positive (Bonanno et al. 2007; Guil et al. 2020).

Especially, people over the age of 65 seem to be more resilient (Bonanno et al. 2007). Contrary to what can be found in literature, our findings do not match aforesaid observations. Forty-one-point-nine percent of our study population belonged to the group of 65 years or older, suggesting that a large part of our population would happen to have a higher resilience. When comparing the mean resilience score of the over 65-year-old patients’ to the mean resilience score of people younger than 65 years, no significant difference is shown.

On the other hand, it is interesting that a study that especially focused on cancer patients noticed a negative correlation of age and resilience. Here patients with a higher resilience level appeared to be younger than the ones with a lower level (Macia et al. 2020). This implies that the factor of having a cancer diagnosis could play a crucial role in terms of the correlation, since our study population consists of cancer patients and patients with cancer in their history; this explains why results differ from literatures only focusing on the correlation of resilience and age. Together with the consideration that resilience might be stronger connected to the patient’s state of cancer, our finding of no correlation between age and resilience could be explained. Our population naturally consists of rather older patients, which should be more resilient, according to Bonanno et al. (2007) or Matzka et al. (2016). Taking our results of resilience and time since diagnosis into consideration, which shows a negative correlation, one reason might be found why age does not seem to correlate. These two factors could counterbalance each other.

Mentioning resilience and the time since receiving the diagnosis, these terms are correlated negatively in our study. This might point to a decrease in resilience if treatment lasts longer, since the process of treatment often means a strong mental as well as physical burden. Further investigations on the effect of long treatment on resilience could give more detailed information on this.

Above that, “time since diagnosis” means that our questionnaire asked for the first time being diagnosed with cancer; at the time of data acquisition, some patients commented to have gone through relapses, metastases, or received new cancer diagnoses. All these different aspects will undisputedly also significantly influence resilience and mental health in general.

Macia et al. (2020) reported patients during treatment showing higher resilience. Since our study was pursued in oncological centers, where most of our polled patients certainly will be receiving therapy, this factor might be less effectful; nevertheless, the difference of being in palliative cancer treatment after a longer treatment process, in contrast to someone who just recently started therapy, based on a new cancer diagnosis, may influence the patients’ resilience. A patient who is in process of curative treatment and more hopeful to be cured from the disease will approach therapy with a different mindset than a patient who is in palliative treatment elongate the time free of complaints. In respect to possible future studies, it would be helpful to investigate whether a lower resilience can also be brought into context with a shortened overall survival of cancer patients or with a general lower quality of life.

Also, in contrast to the literature, our results do not show a correlation between resilience and gender. Several authors reported male gender coming with a higher level of resilience in case of traumatic events, other chronic diseases, or incisive events (Bonanno et al. 2007; Masood et al. 2016; Portnoy et al. 2018).

Hodes and Epperson (2019) point out that hormonal changes, especially for women in life phases like puberty, pregnancy, and perimenopause, decrease their resilience which would apply to a substantial part of our collective which in part receive treatments interfering with hormonal status as in breast cancer.

In contrast, conscientiousness is reported to be higher in women (Limura and Taku 2018), which may strengthen resilience by better emotion regulation (Vaughan et al. 2019). Thus, female patients may gain resilience. As mentioned, our study could not find a correlation and so summed up no clear statements for gender and resilience can be made, implying that this is certainly not a crucial determining variable that should be focused on primarily.

Evaluating the association of resilience and the marital status, no correlation was found. However, marital status and also religion could also be seen as part of social network/support, which makes it even more interesting that apparently no significant correlation is attested. Bonanno et al. (2007) highlight the link between a low social support and weaker resilience levels. At the same time, Zhang et al. (2017) show the strong interaction between a well-established social support and higher levels of resilience. Based on our data, we can only say that the marital status itself is not related to resilience and thus probably not to be equated with social support. Schulz and Schwarzer (2004) report that social support can be divided into many types and spousal support is only one of it. Spousal support does not give any information about the legal marital status, reinforcing that the status itself is not a factor to be considered helpful in screening patients at risk.

In line with our data, Reguera-García et al. (2020) have shown that there is no significant correlation between resilience and religion. However, religion might still play an important factor for mental health in general (Weber and Pargament 2014). This does not necessarily mean a specific religious affiliation, but implies that other factors such as the faith in oneself with, for example self-efficacy and sense of coherence as surrogates, which help patients cope mentally.

In addition, we found a positive correlation for resilience and the financial status. This is in line with the findings by Friborg et al. (2005) who have shown that a lower income goes along with a worse mental health status. Moreover, health issues with severe diseases even in a country with high standards of health care imply financial burdens for patients. Accordingly, Portnoy et al. (2018) reported that a higher resilience is seen in patients a higher income.

We have shown a weak correlation between resilience and education. According to a study among Indian women by Fahey et al. (2016), a higher level of education marks for better coping skills and stress management as well as problem solving, which goes along with our findings in our study. Also, Bonanno et al. (2006) revealed that a higher level of education is associated with greater resilience.

### Resilience and lifestyle

Our data show a significant association between dietary habits and resilience which is in line with other studies’ findings (Owen and Corfe 2017).

A higher resilience enables patients to adhere to a healthy diet. Moreover, they are likely to be empowered through positive feedback from themselves as well as from their environment.

Quite similar, we have shown a positive association between resilience and physical activity.

A study among college students (San Roman-Mata et al. 2020) focusing on physical activity and factors such as general resilience and psychological distress showed a higher physical activity goes along with higher average values for resilience. Physical activity may strengthen the patients’ physical and mental resources as being more physically active up to doing moderate exercises and sports has a positive impact on the immune system (Schmidt et al. 2017). Our patients were asked to elaborate on the frequency of daily activity rather than the intensity, so that the extend of influence by physical activity on the patients resilience cannot fully be clarified. Nevertheless, the average of our patient’s daily activity lies above the recommendation (at least 30 min, 3–5 times a week moderate activity, or more intense activity 75 min per week with about 31–60 min a day, both before and after receiving the diagnosis) (Beckmann 2021; BZgA 2016). This finding adds to Eicher et al. (2015) and Schumacher et al. (2014) that the relation can be seen both ways; a higher physical activity and better physical functioning can affect resilience positively.

Findings by Ristevska-Dimitrovska et al. (2015) report that patients with a lower level of resilience have a more negative body image and report more severe symptoms as well as more negative future perspectives.

### Resilience and psychological factors

To the best of our knowledge, there are no data on correlation of resilience and satisfaction with life as an individual variable in cancer patients, but mainly in the context with quality of life. We found a significant positive correlation of resilience and general life satisfaction.

Resilience includes the restoration to a stable psychological status and being satisfied with one’s life can be seen as part of a stable mental status, implying that supporting the patients’ satisfaction with life will affect resilience as well. One can imagine that someone who displays a low life satisfaction will be less likely to (actively) restore and cope with dramatic events.

With just few-to-no data on satisfaction with life and resilience, more data are published on resilience and quality of life. Macia et al. (2020) point out that resilience is found to be negatively correlated with the part of quality of life that is influenced by physical components, whereas resilience and mental components of quality of life seem to be positively related. This contains a wider array of components besides satisfaction with life. Our finding could be helpful in practice, as this is a variable that can be screened for easily without the need of detailed questioning and evaluating.

We report a strong positive correlation between self-efficacy and resilience. Agreeing, a study in adolescents shows that resilience is an important factor in coping and that the way people use their inner resources has strong influence on the outcome (Hamill 2003). Having a strong self-efficacy helps patients to master difficult situations successfully. People that are self-efficacious have the ability to be more positive and reject negative thoughts rather than others (Hamill 2003). In the context of highly complex cancer treatments, self-efficacy is an important trait to improve self-management and cooperation with the physicians, increase adherence, and improve the management of side-effects.

Moreover, also resilience and sense of coherence are associated. Also, Reguera-García et al. (2020) observed a moderately high correlation between total coherence and resilience. A higher sense of coherence helps to better cope with threatening situations as the diagnosis of cancer, of a relapse and many treatment situations and to actively work on improving their health outcomes (Fok et al. 2005).

One main point of resilience is the ability to cope with and restore from challenging situations; accordingly, the construct of sense of coherence includes coping with events on the basis of manageability, comprehensibility, and meaningfulness (Fok et al. 2005). This shows the close connection between resilience and a person’s sense of coherence and, therefore, the importance of both in the course of a patient’s cancer diagnosis and treatment.

Schafer et al. (2020) also emphasize the importance of strengthening people’s sense of coherence to become more resilient toward challenging situations. These findings support our results that resilience and sense of coherence have the strongest correlation among the study.

## Conclusion

Resilience is an important individual factor in cancer care.

For physicians and nurses, two aspects are important. First, to detect patients who may have a low resilience. A low resilience can be associated with factors as an instable financial status, poor dietary habits, or a combination of the aforementioned.

Second, increasing or at least stabilizing resilience should be integrated into routine cancer care. This includes aspects such as support by psychological support to build self-consciousness, practice positive thoughts, and coping with setbacks, as well as setting achievable goals to keep motivation up and also taking influence on for example perceived sense of coherence. To detect patients with more risk factors, a screening for those at risk before starting therapy appears to be an effective method.

Guided by the individual risk factors, additional therapy concepts may be considered, as for instance psychological therapy, psychoeducation, nutritional advice, as well as encouragement into sports and being active. From our study, it seems that especially for diet and activity, patients are already sensitized and it could be an effective starting point for strengthening resilience directly or indirectly. Due to the fact that for most patients, dietary or activity level adaptions would not be totally new it might be easier to integrate it even more into their everyday life but also achievements are made faster and the individual person might be encouraged.

This way, self-efficacy, life satisfaction, and other psychological factors could be enhanced and indirectly impacting resilience. In addition to that, also the common effects of being active and following a healthy diet are beneficial for the patient and therapy.

For the purpose of promoting or stabilizing resilience, it appears to be helpful to introduce a basic program where individual adaptions according to the patient’s status and needs can be made. In fact, from our data, one might argue that an affirmatory information program on healthy lifestyle might be able to further resilience during and after cancer treatment. To obtain more evidence on this topic, resilience could be chosen as endpoint in studies including lifestyle elements. Moreover, a further assessment of the association between adherence to a healthy lifestyle and quality of life would be helpful.

To develop guidance notes for treatment and cancer patient activation, it would be advisable to consider the patient’s individual educational level and screen for cancer patients that might fall out of alignment in terms of a less favorable educational background.

It seems to be beneficial to strengthen patients in resilience, decelerate the remission of resilience that seems to go hand in hand with a longer time since receiving the diagnosis, and support them in enhancing psychological resilience over the time of treatment and after. The combination of a low resilience and low income makes the patients a more vulnerable group, and hence, they should be screened for this predisposition.

All the different psychological factors are very close related and cannot be solitarily affected or altered. Hence, by strengthening, for example, self-efficacy, resilience is inevitably impacted as well. This relation makes more heterogenic treatments and approaches, for the individual patient possible, depending on their strengths and weaknesses.

Moreover, the patient can actively take part in their therapy or conversely by actively integrating the patients in their treatment a positive impact on the different constructs can be achieved.

To resume our findings and examined literature on resilience, it can both be seen as a stable resource (Ristevska-Dimitrovska et al. 2015) or a factor that can be altered, trained and is dynamic. This study encourages both conceptions depending on the considered variable.

## Limitations

Concerning the type of cancer, our study population of 394 participants split into 10 different types of cancer, which made it difficult to further investigate on possible relations of resilience and the individually different cancer types.

The main limitation of cross-sectional studies is that due to the fact of collecting data at a certain point of time, we cannot establish a cause–effect relation. Also, a unrepresentative timing might be confounding results, depending on timing the patient might be in a state of mind or position where situations are likely to be evaluated more negatively or positively, for example right after receiving discouraging news.

For several items, our questionnaire does not provide detailed information. For example, we asked the participants to state which religion they adhere to. From this, we may not conclude whether they are actively involved in their religious community. Moreover, we did not ask for more detailed data on cancer treatment. Accordingly, we do not know whether patients are under current treatment or survivors. Also, the type of treatment is not elaborated on, which nevertheless could be a variable to be taken into consideration by possible follow-up studies. For the AFHC, we cannot distinguish whether the food habits have changed since the diagnosis.

## Data Availability

The datasets that were generated and analyzed during this study are available from the corresponding author upon reasonable request.
